# Robot Cookies – Plant Cell Packs as an Automated High-Throughput Screening Platform Based on Transient Expression

**DOI:** 10.3389/fbioe.2020.00393

**Published:** 2020-05-05

**Authors:** Benjamin Bruno Gengenbach, Patrick Opdensteinen, Johannes Felix Buyel

**Affiliations:** ^1^Fraunhofer Institute for Molecular Biology and Applied Ecology IME, Aachen, Germany; ^2^Institute for Molecular Biotechnology, RWTH Aachen University, Aachen, Germany

**Keywords:** *Agrobacterium tumefaciens*, automated transient expression, design of experiments, plant molecular farming, rapid protein synthesis, small-scale production

## Abstract

The high-throughput screening of recombinant protein expression is advantageous during early process development because it allows the identification of optimal expression constructs and process conditions. Simple screening platforms based on microtiter plates are available for microbes and animal cells, but this was not possible for plants until the development of plant cell packs (PCPs), also known as “cookies,” which provide a versatile and scalable screening tool for recombinant protein production. PCPs are prepared from plant cell suspension cultures by removing the medium and molding the biomass. PCPs can be cast into 96-well plates for high-throughput screening, but the manual handling effort currently limits the throughput to ∼500 samples per day. We have therefore integrated the PCP method with a fully automated laboratory liquid-handling station. The “robot cookies” can be prepared and infiltrated with *Agrobacterium tumefaciens* by centrifugation, minimizing operator handling and reducing the likelihood of errors during repeated runs, such as those required in a design of experiments approach. The accumulation of fluorescent protein in the cytosol, apoplast, endoplasmic reticulum or plastids is easily detected using an integrated plate reader, reducing the inter-experimental variation to <5%. We also developed a detergent-based chemical lysis method for protein extraction in a 96-well format, which was adapted for automated downstream processing using miniaturized columns allowing subsequent protein analysis. The new automated method reduces the costs of the platform to <0.5 € per PCP infiltration (a saving of >50%) and facilitates a five-fold increase in throughput to >2500 samples per day.

## Introduction

The production of recombinant proteins for human and animal health is a growing market dominated by antibodies, vaccines, replacement enzymes, and modulators such as hormones, growth factors and cytokines (Gifre et al., 2017; Lagassé et al., 2017; Spiegel et al., 2018; Fischer and Buyel, 2020). These prophylactic, diagnostic and therapeutic proteins are mostly produced in microbial cells, such as *Escherichia coli* or *Saccharomyces cerevisiae*, or in mammalian cell lines such as Chinese hamster ovary (CHO), murine myeloma (NS0, Sp2/0) or the human cell lines HEK293 and Per.C6 (Ferrer-Miralles et al., 2009; Li et al., 2010; Dumont et al., 2016). All of these platforms have limitations in terms of process economy, product safety, and the ability to produce proteins that are toxic to the host. They also require a sterile environment and sophisticated process controls. The transient expression of proteins in plants infiltrated with *Agrobacterium tumefaciens* can tackle these issues because the production of plant biomass is cost-efficient and scalable, and the plants act as self-contained bioreactors (Buyel and Fischer, 2012; Buyel, 2018). Furthermore, plants do not support the growth of human pathogens, they can be engineered to carry out authentic post-translational modifications (Jansing et al., 2019), and they can accumulate proteins such as toxins that kill mammalian cells (Gamerith et al., 2017; Gengenbach et al., 2019). Transient expression in plants is also rapid, with even large-scale experiments requiring only weeks from gene to product (Garabagi et al., 2012; Shoji et al., 2012; Sainsbury and Lomonossoff, 2014). In contrast, the development of mammalian cell lines takes many months even before production commences. Finally, recent advances in the downstream processing of plant biomass have resulted in processes that closely resemble those established for other systems in terms of costs and unit operations (Buyel, 2015).

Automated services to handle expression, product analysis and purification have been developed for microbial and mammalian cells, and these facilitate high-throughput screening to identify optimal constructs and expression conditions (Doyle, 2009; Wang et al., 2009; Xiao et al., 2010; Dortay et al., 2011; Kwon and Peterson, 2014; Bos et al., 2015; Wang et al., 2018). There are fewer options in plants, although multiplex experiments have been described using leaf disks and a recently published platform known as plant cell packs (PCPs), in which cells are separated from the growth medium and molded into a compact mass (a “cookie”) that shares many features with plant tissues (Piotrzkowski et al., 2012; Rademacher et al., 2019). PCPs are versatile because they can be prepared from various plant cell suspension cultures, including the widely used tobacco (*Nicotiana tabacum*) BY-2 cell line, and the porous structures can be infiltrated with *A. tumefaciens* to trigger transient protein expression within 3–5 days. Even so, both leaf discs and PCPs have a limited throughput because substantial manual work is required during their preparation, increasing costs and the likelihood of handling errors.

Unlike leaf disks, the preparation and handling of PCPs in microtiter plates can be automated using commercially available laboratory robotics (Rademacher et al., 2019). Furthermore, PCPs can be generated during continuous plant cell fermentation (Blessing et al., 2015) thus reducing the intra-batch and inter-batch coefficients of variation (CV) for protein levels to 5 and 10%, respectively, which is far better than the 10–50% reported for leaf disks (Buyel and Fischer, 2012; Piotrzkowski et al., 2012). PCPs therefore offer a better prediction model for transient protein expression in plants based on suitable correlation factors that account for differences in cultivation conditions, and these factors can be determined by machine learning.

Here we describe the automation of PCP preparation and handling, which facilitates the rapid and cost-effective screening of recombinant protein production using “robot cookies.” Automation increases the throughput of the process to ∼300 infiltration samples per hour. Our automated method includes the cultivation of bacteria and plant cells, the casting of PCPs into 96-well plates, infiltration with bacteria, and protein extraction. Each step is compatible with design of experiment (DOE) strategies to allow the multiplex screening of different constructs and expression conditions in a short experimental timescale.

## Materials and Methods

### Plant Expression Vectors and Bacterial Cultures

The pTRA vector, a derivative of pPAM (GenBank AY027531), was used as the plasmid backbone for all expression constructs. The expression of DsRed, a fluorescent marker protein, was controlled by the double enhanced Cauliflower mosaic virus 35S promoter, 3′ untranslated region (UTR) and polyadenylation signal (Supplementary Table S1). Plasmids were propagated in *E. coli* cultured in lysogeny broth (5 g L^–1^ yeast extract, 10 g L^–1^ tryptone, 10 g L^–1^ sodium chloride, pH 7.0) supplemented with 100 mg L^–1^ ampicillin at 37°C, and were transferred to *A. tumefaciens* strain GV3101:pMP90RK by electroporation (Main et al., 1995). *A. tumefaciens*, used for transient expression, was inoculated from 50% [v v^–1^] glycerol stocks at an optical density at 600 nm (OD_600__*nm*_) of 2.5 into liquid peptone agrobacterium medium 4 (PAM) (Houdelet et al., 2017) supplemented with 50 mg L^–1^ carbenicillin, 25 mg L^–1^ kanamycin and 25 mg L^–1^ rifampicin. To measure the OD_600__*nm*_, *A. tumefaciens* samples were diluted 1:20 in phosphate buffered saline (PBS; 137 mM sodium chloride, 2.7 mM potassium chloride, 10 mM disodium hydrogen phosphate, 1.8 mM sodium dihydrogen phosphate, pH 7.4) in a total volume of 200 μL. To correct for any path-length variations due to cell concentration effects, measurements in a CellStar Clear F-Bottom microplate (Greiner Bio-One, Kremsmünster, Austria) were normalized to standard microtiter–cuvette values using a second degree polynomial correction curve with coefficients b_2_ = 1.568, b_1_ = 2.943 and b_0_ = –0.0003.

### Automated Subcultivation

The *A. tumefaciens* glycerol stocks were manually inoculated into 500 μL PAM per well in a Riplate 96 deep-well round-bottom plate (Ritter, Schwabmünchen, Germany) and covered with a gas-permeable membrane (water vapor transmission rate = 4200 g m^2^ d^–1^). The pre-culture was incubated on a Thermoshake heated orbital shaker (Inheco, Planegg, Germany) at 1000 rpm (4 mm eccentricity) and a set temperature of 30°C (in-well temperature = 27.9 ± 0.5°C, *n* = 2). After incubation for 24–48 h, the OD_600__*nm*_ was automatically measured using a the 96 head with 235 μL sterile clear standard tips at a 50 μL s^–1^ and the volumes of PAM and pre-culture required for the 500 μL main culture with a starting OD_600__*nm*_ of 0.1 were automatically calculated and combined in a fresh deep-well plate. The main culture was incubated for a further 24 h before use.

### Automated Normalization of Infiltration Suspension

The main culture was pelleted at 3700 × g and the supernatant was removed at 75 μL s^–1^ using 1000 μL conductive wide bore tips. The cells were resuspended in 200 μL of infiltration buffer by 30 cycles of aspiration and dispensing at 150 μL s^–1^ using the same head and tips as before, while the plates were agitated at 1000 rpm on a Thermoshake heated orbital shaker. The OD_600__*nm*_ of the concentrated cell suspension was measured as described above using a second degree polynomial correction curve with coefficients *b*_2_ = –4.624, *b*_1_ = 4.525, and *b*_0_ = –0.002. Based on a user-defined total sample number and target OD_600__*nm*_, the appropriate cell suspension and infiltration buffer volumes were calculated and combined to produce a working cell suspension.

### Transient Expression in PCPs

Cells from a continuous suspension culture of the *N. tabacum* BY-2 cell line (100 g wet biomass L^–1^, packed cell volume = 30–40% [v v^–1^]) were concentrated twofold by sedimentation for 40 min followed by the removal of liquid medium (Blessing et al., 2015). PCPs were cast from 300 μL of concentrated cell suspension into 96-well Receiver Plates with a membrane pore size of 50 μm (Macherey-Nagel, Düren, Germany) or in the automated setup 96-well AgroPrep Advance PP/PE 30–40 μm filter plates (Pall, Dreieich, Germany) using 1000 μL conductive wide bore tips at an aspirate and dispense speed of 150 and 300 μL s^–1^. Excess liquid was removed either by applying a vacuum of 80 kPa for 10 s on a chromabond vacuum manifold (Macherey-Nagel) or by centrifugation at 100–3200 × *g* for 1–10 min according to the statistical DOE model. *A. tumefaciens* cells were pelleted by centrifugation at 1300–3800 × *g* for 1–5 min in 96-well deep well plates or at 14,000 × *g* for 1 min in 2-mL reaction tubes. The bacterial pellet was then resuspended in infiltration buffer [0.5 g L^–1^ Murashige-Skoog type medium M0221 (Duchefa Biochemie, Haarlem, Netherlands), 50.0 g L^–1^ (146 mM) sucrose, 2.0 g L^–1^ (10 mM) glucose monohydrate, 0.0392 g L^–1^ (0.2 mM) acetosyringone (Carl Roth, Karlsruhe, Germany), 2.928 g L^–1^ (15 mM) 2-(*N*-morpholino) ethanesulfonic acid (Carl Roth), pH 5.6] and adjusted to an OD_600__*nm*_ of 0.05–0.5. Infiltration of the PCPs was achieved by applying 100 μL of the infiltration suspension (1.7 mL g^–1^ PCP) at a rate of ∼50 μL s^–1^ using the 96 head with 235 μL clear standard tips onto the surface of the PCP followed by incubation for 60 min at 22°C. The PCP plates were then sealed using gas-permeable membranes with vapor transmission rates of 1 or 450 g m^–2^ d^–1^ (4titude, Wotton, United Kingdom), 700 g m^–2^ d^–1^ (Diversified Biotech/Sigma, St Louis, MO, United States), or 4200 g m^–2^ d^–1^ (Macherey-Nagel as well as Thermo Fisher Scientific, Waltham, MA, United States; two membranes with the same nominal permeability were tested) and optionally covered with a universal microtiter plate plastic lid. The plates were incubated for 3–4 days at 26°C and 80% relative humidity over a standard 96-well microtiter plate with 0–100 μL of deionized water per well, or inverted over a 1-well reservoir with 150 mL of deionized water and 150–200 mL headspace per plate (Supplementary Table S2). Alternatively, plates were incubated in an inverted position without a lid or membrane over the aforementioned reservoir.

### Protein Extraction From PCPs

PCPs were supplemented with 3 mL of standard extraction buffer (40 mM disodium hydrogen phosphate, 10 mM sodium dihydrogen phosphate, 10 mM sodium metabisulfite, 500 mM sodium chloride, pH 8.0) per gram fresh biomass and then lysed. Manual lysis was carried out either in a rack of 1.2-mL collection microtube strips (Qiagen, Hilden, Germany) using an MM 300bead mill (Retsch, Han, Germany) at 28 Hz for 2 × 3 min with one 3-mm steel bead per well, or the PCPs were transferred to 1.5-mL reaction tubes and lysed with an electro-pistil for 15 s.

Alternatively, for automated lysis, PCPs were transferred from the 96-well AgroPrep Advance filter plates into standard 96-well U-shaped assay plates by inverted centrifugation at 1800 × *g* for 1 min using a custom plate adapter for sandwich centrifugation. The PCPs were then disrupted in lysis buffer (0.0001–1.0000% [m v^–1^] sodium dodecylsulfate (SDS), 0–50 mM ethylenediaminetetraacetic acid (EDTA), and 10–50 mM tris(hydroxymethyl)aminomethane (Tris) or phosphate, pH 6.0–9.0) by simultaneous pipetting (150 μL lysis buffer per PCP with 50–150 μL s^–1^, 10–50 cycles) and shaking (100–1000 rpm, 2 mm eccentricity). Optionally, we transferred 150 μL of a previously generated PCP suspension to standard 96-well PCR plates and incubated them for 20–120 min at 22–80°C. The final buffer composition and protocol settings were selected based on a DOE optimization approach (Supplementary Table S3).

### Quantitation of Protein and BY-2 Cell Mass, PCP Mass Ratios and PCP Mass Loss

The total soluble protein (TSP) concentration was determined using a modified standard or detergent-compatible Bradford protein assay (Thermo Fisher Scientific) in a microtiter format (5 μL sample and 195 μL Bradford solution using six dilutions of bovine serum albumin from 0.125 to 2.000 g L^–1^ for quantitation). DsRed fluorescence on the PCP surface or in the extract was measured at an emission wavelength of 585 nm after excitation at 559 nm using an Enspire plate reader (PerkinElmer, Waltham, MA, United States). The DsRed concentration in extracts was calculated based on linear curve fitting (5.0–200.0 mg L^–1^) using purified DsRed as a quantitation standard.

The protein composition of each sample was analyzed by lithium dodecylsulfate polyacrylamide gel electrophoresis (LDS-PAGE) using NuPAGE 4–12% Bis-Tris protein gels under reducing conditions (Thermo Fisher Scientific). The gels were stained with Coomassie Brilliant Blue and the band pattern was analyzed using AIDA Image Analysis software (Elysia-raytest, Straubenhardt, Germany).

Following the resuspension of PCPs, the absorbance of the single-cell suspension was measured at 290 nm because this produces the strongest adsorption signal in the 230–1000 nm spectrum and shows a linear correlation with BY-2 cell mass in suspension (0.03–0.15 kg L^–1^). The concentration of resuspended cells was calculated based on a linear standard curve (Supplementary Figure S1). For experiments that required the determination of PCP mass ratios, plates were separated into peripheral (*n* = 16) and inner (*n* = 8) well PCPs, which were pooled before analysis (Supplementary Figure S2).

### Fluorescence Microscopy of PCP Sections

XZ-planar mid-sections of ∼0.2–0.5 mm thickness were manually prepared using a razor blade, transferred to microscope slides and moistened with ∼5 μL of PBS immediately before imaging. Fluorescence microscopy was performed using a Leica DMRB fluorescence microscope (Leica Microsystems, Wetzlar, Germany) with a 10 × high-contrast flat-field ocular and 1.6 × air objective (numerical aperture 0.05), and excitation and emission wavelengths of 530 nm and 590 nm, respectively. The exposure time was 1.0 s for white light and 0.53 s for fluorescence imaging. The gain factor was set to 2.1 in all cases. For illustration purposes, a red channel filter mask was applied to fluorescence images and selected areas were superimposed on white-light images using Photoshop CS5 (Adobe, San Jose, CA, United States).

### Design of Experiments Software

We generated and evaluated i-optimal designs using Design Expert v10.0.0.3, build 28 Jan 2016 (Stat-Ease, Minneapolis, MN, United States) for system characterization and optimization (Supplementary Tables S2–S6). Alternatively, pooled data were evaluated using the historical data analysis function of the same software. For i-optimal designs, the number of runs was adjusted to obtain a fraction of design space coverage of ≥0.95 for an effect size twice as large as the standard deviation (α = 0.05). Significant model factors were automatically identified by backward selection from a reduced cubic or quadratic base model based on a *p*-value threshold of 0.05.

### Statistical Testing

The Kolmogorov–Smirnov test (Berger and Zhou, 2014) was used to ensure normal data distribution, then a two-sample *F*-test (Box, 1953) was applied to test for equal variances between samples. Finally, either a two-sample two-sided Student’s *t*-test (equal variances), or Welch’s *t*-test (unequal variances) was used to determine the differences between sample means (α = 0.05 in all cases).

### Automation

A JANUS G3 laboratory liquid handling station with an extended Gripper (PerkinElmer) controlled by WinPREP Automation Software v4.1.3 was used for automated PCP generation. The system was integrated with vacuum manifold labware (PerkinElmer), an Enspire plate reader (PerkinElmer), a LabchipGXII protein characterization system (PerkinElmer), an On-Deck Thermo-Cycler 96 (Inheco), a Thermoshake heated orbital shaker, and a Rotina 380R Robotic centrifuge (Hettich, Tuttlingen, Germany).

## Results and Discussion

### Definition of Quality Criteria, Targets and Process Steps

**FIGURE 1:**
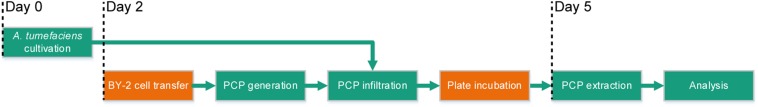
Process flow of the automated PCP process for high-throughput screening applications. The green boxes show the steps of the process that were automated (cultivation of *A. tumefaciens*, PCP generation and infiltration, PCP extraction and analysis) and the orange boxes show the steps that required manual intervention (BY-2 cell transfer and plate incubation after infiltration) although these steps should be easy to automate at a later stage. Days 0–5 indicate the chronology of the subroutines.

The full potential of PCPs (Rademacher et al., 2019) as a transient expression screening platform can only be realized if the method is compatible with automated liquid handling and thus achieves repeatable results in a high-throughput and cost-effective manner. We therefore set out to automate the cultivation of bacterial cells, the casting of plant cells into PCPs, bacterial infiltration and the extraction of recombinant proteins using laboratory robotics, aiming to satisfy two quantitative benchmarks: (1) an overall coefficient of variation (CV) below 5% between replicates; and (2) a cost reduction from ∼1.0 € per infiltrated PCP in the manual process to <0.3 € per PCP in an automated process with a batch size of 10 microtiter plates. Due to space constraints, the automated process did not include BY-2 cell transfer from continuous fermentation or the incubation of PCP plates after infiltration, but these steps can easily be incorporated at a later stage by introducing simple transfer and movement commands (Figure 1).

### Cultivation of *A. tumefaciens* in a 96 Deep-Well Format Is Reproducible and Allows Automated Sample Processing

(1) $$y=\frac{a}{1+e^{-k\,\left(x-x\,c\right)}}$$

Where *x* is the independent variable (here: cultivation time), *y* is the dependent variable (here: OD_600__*nm*_), *a* is the maximum value, *k* is the steepness and *xc* is the midpoint.

Transient protein expression in plants and PCPs is influenced by the number of *A. tumefaciens* in the infiltration suspension, typically represented as OD_600__*nm*_ (Buyel and Fischer, 2012; Rademacher et al., 2019), as well as the physiological state of the bacterial cells (Gelvin, 2006). Uniform bacterial growth is therefore important and, if not a parameter to be optimized, the OD_600__*nm*_ should be kept constant during high-throughput screening in 96-well plates to avoid effects on product accumulation and misinterpretation of the corresponding results.

**FIGURE 2:**
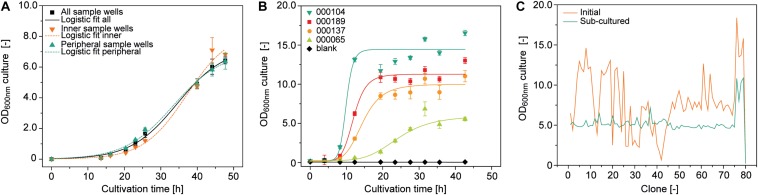
Growth of *A. tumefaciens* cultivated in a 96-well deep plates on the robotic platform. **(A)** Logistic fit (**Equation 1**) (adj. *R*^2^ = 0.996) to growth data from 92 (all sample wells, adj. *R*^2^ = 0.984), 6 (inner sample wells, adj. *R*^2^ = 0.994) or 32 replicates (peripheral sample wells) of *A. tumefaciens* clone 000101 inoculated with an OD_600__*nm*_ of 0.01 from a single culture. **(B)** Logistic fit to growth data of *A. tumefaciens* 000104 (adj. *R*^2^ = 0.97), 000189 (adj. *R*^2^ = 0.98), 000137 (adj. *R*^2^ = 0.98), 000065 (adj. *R*^2^ = 0.97) (*n* ≥ 23) automatically and individually inoculated with a starting OD_600__*nm*_ of 0.1 from pre-cultures with starting OD_600__*nm*_ values of 3.7, 1.7, 2.1, and 4.5, respectively. **(C)** Optical densities of 79 individual *A. tumefaciens* clones carrying different vectors (Supplementary Table S1) after 48 h (orange) and after an additional 24 h following automated sub-culturing step (green). The culture volume per well was 500 μL in all cases, with shaking at 1000 rpm and 4 mm eccentricity.

OD_600__*nm*_ is a function of bacterial growth, which in turn depends strongly on the temperature. We therefore investigated temperature homogeneity throughout a plate with a set point of 30.0°C on a Thermoshake heated orbital shaker and measured the mean ($\bar{x}$) temperature ± standard deviation (SD) in different wells. The temperature of the peripheral wells (H12) was 27.5 ± 0.1°C whereas that of the inner wells (D6) was 28.4 ± 0.1°C (15 min measuring interval over 8 h after 1 h equilibration, *n* = 33). We inoculated the same type of plate with a low starting OD_600__*nm*_ of 0.01 to increase the time required to reach stationary phase because this should increase potential effects of temperature heterogeneity on final OD_600__*nm*_ and therefore facilitate their identification. We observed a ∼7% higher final OD_600__*nm*_ in the inner wells (*n* = 4) compared to the peripheral ones (*n* = 32), with an overall OD_600__*nm*_ of 6.5 ± 0.2 and a CV of 10.2 ± 4.2% (*n* = 93) (Figure 2A). This can probably be attributed to accelerated chemical and biological reactions caused by the higher temperatures in these wells (Arrhenius, 1889). However, accurate modeling of *A. tumefaciens* growth near its temperature optimum of 28°C (Lippincott et al., 1981) within a 0.9°C range is complex and requires substantial data, which was beyond the scope of this study and was not investigated further (Huang et al., 2011).

We also evaluated position-independent homogenous bacterial growth by individually inoculating 92 wells randomly selected from a 96-well plate with pre-cultures from four different vectors (Supplementary Table S1) and a starting OD_600__*nm*_ of 0.1. We used an automated protocol suitable for screening experiments, achieving a favorably low average CV of 7.3 ± 5.7% (*n* = 32) for the OD_600__*nm*_ between replicates. However, differences were significant between vectors (two-sample, two-sided Welch *t*-test with unequal variances, *p* = 3.47 × 10^–6^, α = 0.05, df = 8) with an average CV of 65.8 ± 14.4% (*n* = 8) for OD_600__*nm*_ after growth for 48 h (Figure 2B).

These differences can limit the comparability of the PCP screening results by masking variations in protein accumulation levels due to the properties of the protein or expression construct, so we introduced an automated sub-cultivation step that reset the OD_600__*nm*_ to 0.1 after 48 h, and continued cultivation for a further 24 h. Using this method, the CV over a set of 79 different vectors significantly reduced from 45% (OD_600__*nm*_ = 7.9 ± 3.6, *n* = 81) after 48 h to 22% (OD_600__*nm*_ = 5.3 ± 1.2, *n* = 81) after sub-cultivation and 24 h incubation (two sample *F*-test for variance, *p* = 2.29 × 10^–4^, α = 0.05, df = 38) (Figure 2C). Our approach therefore reduced initial heterogeneity by compensating for variations in inoculum fitness, allowing the adaption of newly transformed clones to the culture medium (Casadesús and Low, 2013; Pavlov and Ehrenberg, 2013). We anticipate a CV of <25%, but instead of implementing a rejection threshold we defined a maximum of three automated sub-cultivation cycles in order to accommodate clones that respond slowly or not at all to sub-cultivation due to the aforementioned factors affecting cell growth kinetics. We were able to compensate for any remaining differences in OD_600__*nm*_ during automated preparation of the infiltration working suspension from bacterial stocks and infiltration buffer. In this way, the CV was eventually reduced to 11% (OD_600__*nm*_ = 0.44 ± 0.04, *n* = 64) with an anticipated infiltration target OD_600__*nm*_ of 0.4 (Supplementary Figure S3).

Undetected mutations in the vector backbone may permanently affect the growth rate of affected clones (Thompson et al., 2018). However, we assumed that differences in biomass prior to inoculation caused the heterogeneity between individual clones after 48 h. On one hand, these differences may originate from variations in the fitness of cultures prior to cryopreservation, reflecting the physiological state of individual cells, the pre-culture cell density, or the duration of exposure to the medium and thus the ability of cells to adapt (Malik, 1991; Tenaillon et al., 2016). On the other hand, the number of live cells in the inoculum can be affected by the cooling and warming rates during freeze–thaw cycles and the mechanical stress caused by vigorous pipetting and high-speed centrifugation, thus influencing the lag times and growth rates (Malik, 1991).

### Vacuum-Based Removal of Medium From PCPs Can Be Replaced by Centrifugation to Simplify Automated Handling in a Non-sterile Environment

**FIGURE 3:**
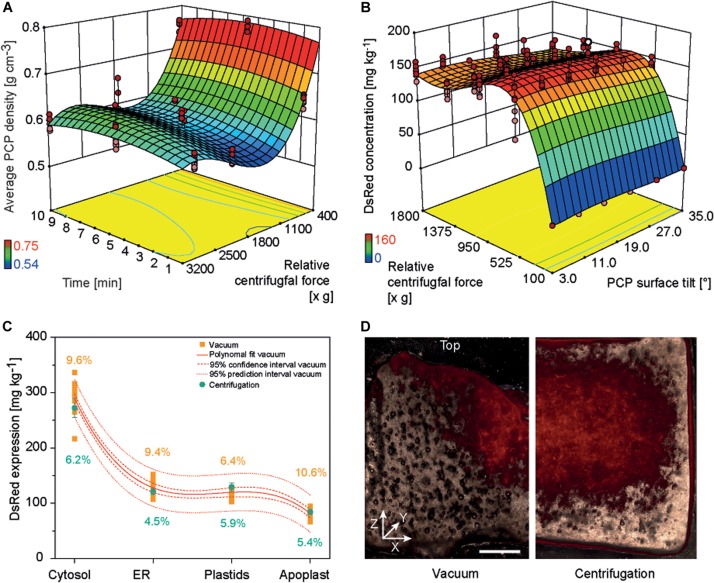
PCP density and DsRed accumulation in PCPs generated by centrifugation or vacuum. **(A)** Response surface of PCP average density as a function of relative centrifugal force (rcf) and treatment time following centrifugation. **(B)** Response surface of DsRed expression as function of rcf and PCP surface tilt angle following the removal of bacterial suspension by centrifugation. **(C)** Comparison of DsRed levels in PCPs generated by vacuum or by centrifugation. **(D)** Fluorescence images (530 nm excitation, 590 nm emission) of PCPs generated by vacuum (left half) and centrifugation (right half) and cut in the XZ (width × height) plane (∼0.5 mm thickness). Horizontal size of scale bar is 1 mm. The vertical white line indicates the center of each PCP. The Z-axis of the coordinate system is directed toward top of the PCP.

Non-sterile conditions are preferable for an automated PCP process because this avoids the cost of large areas of sterile work space to house the required liquid handling station. In the original PCP method, a vacuum was applied manually under sterile conditions to remove cultivation medium or *A. tumefaciens* suspension because non-sterile conditions risked contamination by attracting airborne microbes to the PCP surface (Rademacher et al., 2019). Furthermore, droplets of liquid remaining at the bottom of the filter plate during plate stacking caused the spontaneous run-off of cultivation medium or *A. tumefaciens* suspension due to capillary forces, leading to higher intra-batch and inter-batch variation. We therefore tested liquid removal from PCPs by centrifugation and used a DOE approach to identify conditions yielding low-density PCPs (indicating effective liquid removal without compression of the cell pack, which would affect cell viability and thus the product yield). A centrifugal force of 500–1800 × g for 1–10 min achieved PCP densities below 0.6 g cm^–3^ (Supplementary Tables S4, S6 and Figure 3A).

The casting of PCPs by centrifugation introduced a surface tilt due to the angle of the centrifuge, ranging from 3° in plate columns 6 and 7 to 35° in columns 1 and 12. In a subsequent DOE, we found that increasing the tilt angle reduced the accumulation of model protein DsRed in PCPs by 4–33%, and the highest protein yields were achieved at centrifugal forces of 550–750 × g during the removal of *A. tumefaciens*: 165.2 ± 9.2 mg kg^–1^ (CV 8.1%) for cytosolic DsRed and 58.2 ± 9.2 mg kg^–1^ (CV 9.1%) when targeting the apoplast (Supplementary Tables S3, S6 and Figure 3B). The low yields at higher centrifugal forces were attributed to PCP compression, whereas the even lower yields at lower centrifugal forces were attributed to insufficient liquid removal, which affects PCP viability probably by limiting gas exchange (Rademacher et al., 2019). The yields of DsRed were ∼15% higher at 550–750 × *g* compared to ∼1620 × *g*, which achieved the lowest PCP density. We assume that because some infiltration suspension remained within the PCP, the residual liquid provided additional moisture, preventing desiccation and therefore prolonging the conditions that favor T-DNA transfer, as was previously reported (Gelvin, 2006; Matthysse, 2014).

Because the highest DsRed yields were achieved under conditions that did not meet our predefined target of 5% CV, we used numeric optimization to minimize variation by applying the process capability index criterion to our descriptive model (Kane, 1986). Minimal variation was achieved at a centrifugal force of 1595 × *g*, with CVs of 5.4 and 6.1% for DsRed in the cytosol and apoplast, respectively. This corresponded to a DsRed yield of only ∼14% below the maximum value. We also found that DsRed yields fell within a 95% prediction interval for all four cellular compartments using the centrifugal method. Importantly, the centrifugation method significantly reduced the average CV for all compartments from 9.0 ± 1.8% (*n* = 4) to 5.5 ± 0.7% (*n* = 4) based on an independent samples two-sided *t*-test with equal variances (*p* = 0.012, α = 0.05, df = 6) (Figure 3C). Microscopic analysis revealed that DsRed was homogenously distributed following centrifugation but concentrated at the PCP surface and central region along the vertical axis following vacuum aspiration (Figure 3D). We propose that the small, central outlet at the bottom of each well on the filter plate caused the channeling of liquids under vacuum, which probably concentrated the exposure of plant cells to the bacteria at the surface and in the central region of the PCP. Furthermore, we observed a central indentation on the top surface of each PCP surface when using the vacuum method, which probably reflects the higher packing density of BY-2 cells in that region and thus a higher fluorescence-to-volume ratio. In contrast to the negative impact of increased cell density on protein accumulation described above, this spatially limited compaction of the cells would not have a significant effect on gas exchange and thus did not reduce the fluorescence intensity. The indent may also trigger the tendency of PCPs to form a central crack when prepared by the vacuum method, ultimately reducing recombinant product yield (Supplementary Figure S4) (Rademacher et al., 2019). We therefore regard the switch from vacuum to centrifugation as successful and used the latter for all subsequent experiments. Furthermore, centrifugation may replace vacuum infiltration in case of low or medium throughput applications as well as during manual PCP preparation to exploit the benefits of the former method for example in terms of increased repeatability and reduced contamination risk.

### Inverted Incubation of PCP Plates Prevents Uneven Desiccation and Reduces Signal Variance

Having reduced the CV for recombinant protein levels to ∼6% (still above our threshold of 5%), we looked for additional sources of variability in the system. We found that the PCP mass was only 0.062 ± 0.001 g (*n* = 8) in the automatable low-profile filter plates (AgroPrep Advance) which reduced the volume-to-surface ratio by a factor of ∼1.7 compared to high-profile Receiver Plates (0.246 ± 0.006 g, *n* = 18) assuming a cylindrical PCP shape and diameters of 6.0 and 8.3 mm, respectively (Supplementary Figure S5 A). This lower ratio can risk PCP desiccation, especially at the plate edges, inhibiting protein accumulation and ultimately increasing the intra-batch variation. We therefore evaluated the effect of membranes with different water vapor transmission rates (WVTRs) ranging from 1–4200 g m^2^ day^–1^, the installation of a water reservoir underneath the PCP plates (150 μL per well – 150 mL one well), or regular microplate lids on DsRed accumulation, TSP levels and PCP desiccation in a DOE approach (Supplementary Tables S1, S6). We used the post-incubation mass ratios between the inner and peripheral PCPs of a plate to indicate heterogeneity. Alternatively, we inverted an unsealed PCP plate and placed it over a water reservoir with 150–200 mL headspace, which we termed “inverted incubation” (Supplementary Figure S5 B).

We defined a minimum relative post-incubation PCP mass ≥0.7 g g^–1^ with 0.64 g g^–1^ relative water content (RWC) as an acceptable threshold after a 3-day incubation-phase because the average RWC of untreated *N. tabacum* BY-2 cells was 0.94 g g^–1^ whereas 0.65–0.70 g g^–1^ RWC has been described as drought stress for tobacco (Rizhsky et al., 2002). We also aimed for a DsRed yield that was more than 95% of the level achieved using the original membrane-sealed setup as well as a CV ≤ 5% for recombinant protein accumulation across all wells of a plate. None of the membranes achieved all goals (Supplementary Tables S1, S6), but the inverted incubation strategy met the criteria, achieving a relative post-incubation PCP mass of 0.7 g g^–1^, an inner-to-peripheral PCP mass ratio of 0.9 g g^–1^ and a relative DsRed expression level of 0.98 AFU AFU^–1^ with a CV of 4.6% (Table 1).

**TABLE 1:** Comparison of inverted with membrane-sealed plates in terms of PCP mass homogeneity, mass loss and recombinant protein accumulation after three days of incubation.

Incubation type [–]	Membrane WVTR [g m^–2^ d^–1^]	Water per well in reservoir [mL]	Lid [Y/N]	PCP mass homogeneity^*c*^ [g g^–1^]	PCP mass relative to initial^*d*^ [g g^–1^]	Relative DsRed accumulation^*e*^ [AFU AFU^–1^]	*SD*
Inverted	–	150^*a*^	N	0.90	0.71	0.98	0.046
Standard	4200	0.1^*b*^	Y	0.72	0.53	0.98	0.115
Standard	4200	150^*a*^	Y	0.84	0.61	1	0.058
Standard	700	0.1^*b*^	N	0.95	0.42	0.87	0.092
Standard	1	0.1^*b*^	Y	0.99	0.91	0.38	0.114

*^*a*^1 well reservoir; ^*b*^96 well reservoir; Y, yes; N, no; ^*c*^mass homogeneity = $\bar{x}$_(inner__–_*_*n*_*_=__8)_$\bar{x}$^–1^_(peripheral__–_*_*n*_*_=__16)_; ^*d*^mass relative to initial = $\bar{x}$; day 3_(inner__–__peripheral__–_*_*n*_*_=__24)_${\bar{x}}^{-1}$ day 0_(inner__–__peripheral__–__n=24)_; ^*e*^DsRed fluorescence relative to highest measured value (Standard, 4200 g m^2^ d^–1^, 150 mL) (*n* = 40, excitation: 559 nm, emission: 585 nm); AFU, arbitrary fluorescence unit; SD, standard deviation; WVTR, water vapor transmission rate.*

Such a small across-well variance has not been reported before for transient expression in plants, which usually involves a CV of ∼10–50% (Buyel and Fischer, 2012; Piotrzkowski et al., 2012). Similarly, the minimum CV reported previous for PCPs was ∼10% (Rademacher et al., 2019) which is similar to the ∼7% reported for CHO cells in an automated protein (Wang et al., 2018). To our knowledge, the latter was the lowest CV reported for protein expression screening until the experiments reported herein.

Accordingly, all 96 wells of a plate can be used for expression testing, which is an increase of ∼40% compared to an approach that excludes peripheral wells to avoid evaporation or temperature gradient edge effects (Oliver et al., 1981; Lundholt et al., 2003). Our inverted incubation design is compatible with other applications such as hydrogel or hanging drop cultures (Caliari and Burdick, 2016; Shri et al., 2017) and does not require the use of special plates with a surrounding liquid/gel-filled moat (Hartmann and Tacheny, 2017).

### A Low-Profile Plate Format Allows Automation and the Direct Evaluation of Surface Fluorescence Which Correlates With Extract Fluorescence

**FIGURE 4:**
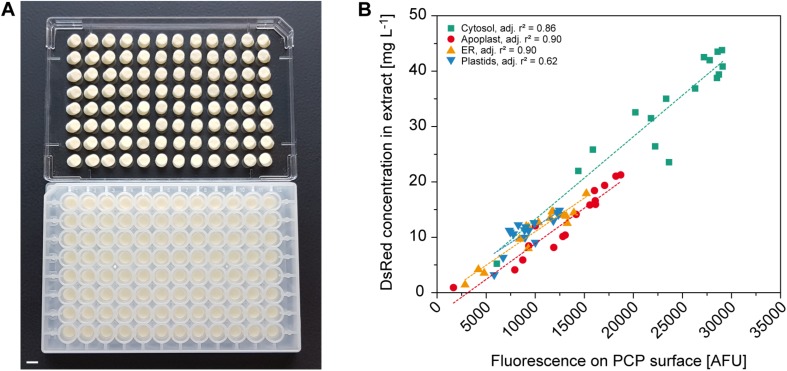
Automated readout of surface fluorescence from PCPs in 96-well plates. **(A)** PCPs in an AgroPrep Advance filter plate after the removal of BY-2 culture medium (bottom) and after inverted centrifugation for clarification of dimensions and pack integrity (top). Scale bar = 5 mm. **(B)** Correlation between fluorescence on PCP surface (excitation = 559 nm, emission = 585 nm) and DsRed concentration in extracts prepared from the same PCPs transiently expressing DsRed in the cytosol, apoplast, endoplasmic reticulum (ER) or plastids 4 days after infiltration (*n* = 16). Dotted lines show linear fit per compartment.

Protein expression screening can be simplified if product concentrations can be determined on the PCP surface because this avoids the need for protein extraction. For example, fluorescent reporter proteins can be used to monitor the effects of regulatory elements such as promoters and 5′ untranslated regions (Fan et al., 2012; Dey et al., 2015), or codon optimization strategies for improved translational efficiency (Mauro and Chappell, 2014), or they can function as an internal quality control tool to track expression over time and different PCP plates. Using *A. tumefaciens* with an OD_600__*nm*_ in the range of 0.001–0.040 for infiltration combined with targeting to the cytosol, apoplast, endoplasmic reticulum (ER) or plastids, we were able to accumulate DsRed to different levels in the PCPs for automated measurement. Data acquisition in high-profile Receiver Plates was impossible because they are not compatible with standard plate readers, but the fluorescence of PCPs was reliably detected in low-profile AgroPrep Advance sterile filter plates (Figure 4A and Supplementary Figure S3). The average compartment-specific adjusted *r*^2^ value of the fluorescence signal was 0.82 ± 0.12 (*n* = 4) compared to fluorescence measured in extracts prepared from the same PCPs using a bead mill (Figure 4B). The only compartment with an adjusted *r*^2^ value below 0.85 was the plastids (0.62), probably due to the limited spread in accumulation levels (only 30% of the range covered by cytosolic expression). We tested this hypothesis by first normalizing all surface and extract fluorescence data (Figure 4B) to the range 0.0–1.0 and then fitting a linear function, yielding f(x) = 0.11 + 0.73 × x, where x and f(x) are the normalized surface and extract fluorescence values, respectively. Based on this function, we next used a numeric simulation tool (GNU Octave, v 5.1.0) to calculate two sets of 10 data points spanning an x-range of either 0.30 or 1.00, resembling the plastid and cytosol data, respectively. We then added random noise with an average of 0.05 to the data points to mimic the variation in the experimental data and fitted linear functions to the 0.30 and 1.00 datasets. We repeated this noise addition and fitting procedure 100 times for both datasets and obtained average adjusted *r*^2^ values of 0.67 ± 0.21 for the 0.30 dataset and 0.97 ± 0.01 for the 1.00 dataset. This closely resembled the experimental adjusted *r*^2^ values for plastid and cytosol fluorescence, and supported our assumption that, with a similar experimental variation, a reduced data range reduces the adjusted *r*^2^. The direct measurement of fluorescence on the PCP surface is therefore a useful tool for rapid screening applications.

### Chemical Extraction Releases 91% of Secreted DsRed Relative to Mechanical Extracts but Require a Correlation Curve to Predict Total Target Protein Levels in the Cytosol, ER and Plastids

**FIGURE 5:**
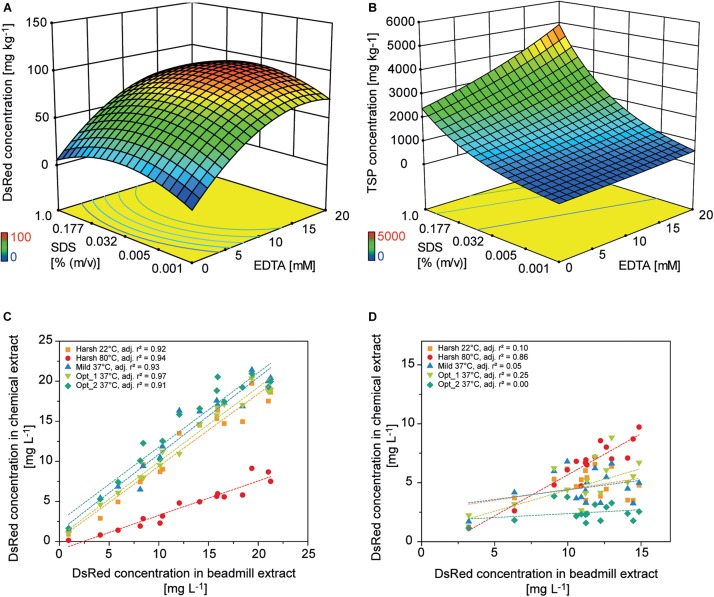
Design of experiments for the optimization of chemical PCP lysis and extraction. Response surface of **(A)** DsRed and **(B)** TSP concentrations in PCP extracts depending on the concentrations of EDTA and SDS [% (m v^– 1^)] in the lysis buffer. Correlation of DsRed concentration after chemical lysis or mechanical extraction from PCPs for **(C)** apoplast-targeted (vector 000102) or **(D)** plastid-targeted products (vector 000104).

Colorless and non-fluorescent recombinant proteins cannot be detected by the analysis of PCP surfaces and product extraction is typically required for quantitation. However, mechanical extraction methods such as bead mills either require extensive manual intervention or robotics equipment that is not part of regular liquid-handling stations. We therefore established a chemical PCP lysis protocol consisting of resuspension by multiple aspiration and dispensing cycles on a shaking platform, the addition of EDTA as a chelating agent to destabilize cell walls, and membrane disruption by SDS (Tsugama et al., 2011). In a first statistical design, we quantified the cell mass in suspension and in a second design we optimized the release of recombinant DsRed and TSP from the cells. We found that cell suspensions reached a plateau after 30 aspiration and dispensing cycles with a shaking speed of ≥800 rpm (4 mm eccentricity) (Supplementary Figure S6 A). In the subsequent cell lysis experiment, DsRed release reached a plateau at an EDTA concentration of >15 mM combined with 0.03% (m/v) SDS, whereas the TSP concentration increased with increasing concentrations of SDS and EDTA (Figures 5A,B). This suggests that low SDS and EDTA concentrations are sufficient to permeabilize cell walls and membranes, releasing soluble proteins, including recombinant DsRed, from the cells (Shehadul Islam et al., 2017), whereas higher chelator and detergent concentrations solubilized an increasing number of membrane-associated proteins (Seddon et al., 2004) resulting in the observed increase in TSP. Modifying the lysis buffer pH in the 7.0–9.0 range had no significant effect on DsRed or TSP concentrations (DOE analysis of variance, α = 0.05), whereas increasing the temperature from 37 to 60°C increased the average DsRed and TSP concentrations by a factor of three (Supplementary Tables S5, S6). This agreed with the lipid solubilization kinetics of SDS, which are known to exhibit the same temperature dependence due to trans-bilayer flip-flop of the SDS molecules (Keller et al., 2006). We then used the descriptive models to identify conditions for high DsRed and TSP concentration (Opt 1) or for high DsRed and low TSP concentration (Opt 2) and compared them with compositions previously suggested for *Arabidopsis thaliana* leaf material (Table 2). Importantly, model protein DsRed was shown to be stable in raw plant extracts for several minutes (Buyel and Fischer, 2014), however, the stability of other recombinant proteins has to be evaluated in order to obtain reliable, quantitative results for accumulation after extraction.

**TABLE 2:** Lysis buffers tested for refined protein extraction from PCPs.

Lysis buffer type	EDTA [mM]	SDS[% (m/v)]	Application	Modified based on
Harsh	50.0	1.000	Maximum lysis	Tsugama et al., 2011
Mild	1.0	0.009	Preservation of tertiary structure	Bhuyan, 2010
Opt 1	3.7	0.022	Highly soluble and TSP	This study
Opt 2	7.3	0.002	Highly soluble, low TSP	This study

*Opt, numerical optimization using DOE descriptive models.*

The predictions were confirmed experimentally and correlated well with a mechanical reference extraction (bead mill) when DsRed was targeted to the apoplast (adj. *r*^2^ = 0.93 ± 0.02, *n* = 5) or ER (adj. *r*^2^ = 0.83 ± 0.11, *n* = 5). Considering all lysis treatments, chemical DsRed release from the apoplast achieved 91 ± 34% (*n* = 80) of the mechanical reference value, which was substantially more than the 40% achieved using an earlier protocol (Rademacher et al., 2019). However, the correlation was poor for plastid-targeted DsRed (adj. *r*^2^ = 0.25 ± 0.35, *n* = 5) unless we used a harsh lysis buffer treatment at 80°C (adj. *r*^2^ = 0.86) (Figures 5C,D and Supplementary Figures S6B,C). We concluded that the chemical lysis method had a limited ability to permeabilize the four membranes separating the plastid-targeted DsRed from the extracellular space and therefore released only a fraction of the product from the biomass. The Opt 2 condition for selective extraction of soluble proteins significantly increased the relative TSP fraction of DsRed from 0.021 ± 0.016 achieved with Opt 1 to 0.047 ± 0.047 in all compartments (independent samples two-sided t-test with unequal variances, *p* = 8.14 × 10^–6^, α = 0.05, df = 126), whereas the absolute DsRed concentration was not significantly affected.

The good linear correlation between the chemical lysis and mechanical reference extractions will facilitate a comparison to earlier results and data from whole plant extracts, and should be verified with other proteins such as monoclonal antibodies (Sack et al., 2015), vaccine candidates (Jones et al., 2013), and enzymes (Mamedov and Yusibov, 2013). In order to improve the compatibility of the lysis buffer with the activity of different proteins and diverse analysis methods, it would be useful to screen for components to replace EDTA and/or SDS, such as non-ionic or zwitterionic detergents like Triton X-100 or CHAPS (Shehadul Islam et al., 2017).

### Cell Culture Quality Monitoring

We monitored the compartment-specific recombinant protein levels by expressing the corresponding DsRed variants on individual PCP plates as an indicator of BY-2 cell culture performance and batch-to-batch reproducibility over a course of 12 months. The relative yields of DsRed in the four compartments varied between individual batches and the absolute accumulation levels fluctuated within 13–100% of the maximum. Both observations indicated that, despite our defined media and cultivation conditions (Blessing et al., 2015), the performance of the cells in terms of recombinant protein accumulation can vary substantially. Genetic modifications such as point mutations and epigenetic changes such as DNA methylation are known to occur in plant cell cultures (Phillips et al., 1994), and may cause unintended somaclonal variation that can manifest in a new phenotype (Miguel and Marum, 2011; Wang et al., 2013). The increasing duration of continuous cultivation is likely to enrich for variants (Côte et al., 2001) and prolonged exposure to the synthetic auxin 2,4-dichlorophenoxyacetic acid can trigger chromosome abnormalities in plant cells (Pavlica et al., 1991), negatively affecting cell fitness and transient protein expression. Accordingly, when the BY-2 fermentation runs providing the cells for PCP preparation were maintained for more than 8 weeks, we occasionally observed a drop in growth rate accompanied by lower DsRed yields.

We therefore defined three quality criteria to quantify the variability in product accumulation and to establish thresholds ensuring only high-quality cell batches were used for expression screening. The first criterion was recombinant protein accumulation for which we established an economically relevant threshold based on plant-derived monoclonal antibody 2G12 that was used in clinical phase I trials and accumulated to ∼30 mg kg^–1^ fresh PCP biomass or ∼8 mg L^–1^ extract under regular conditions (Rademacher, 2012; Ma et al., 2015; Rademacher et al., 2019). The minimal acceptable accumulation of 2G12 due to variability in expression and thus PCP performance was set to 0.4 mg kg^–1^ biomass corresponding to 0.1 mg L^–1^ extract, as this concentration is routinely detected by dot blot assay (Falconar and Romero-Vivas, 2013). Because antibody concentration was not amenable by direct measurement, we used absolute DsRed surface fluorescence as a surrogate, where ∼30 mg kg^–1^ 2G12 corresponded to ∼20 mg kg^–1^ DsRed, which was equivalent to ∼7500 AFU. Accordingly, 0.4 mg kg^–1^ 2G12 corresponded to 0.3 mg kg^–1^ DsRed or ∼100 AFU, which we used as a minimal accumulation threshold.

**FIGURE 6:**
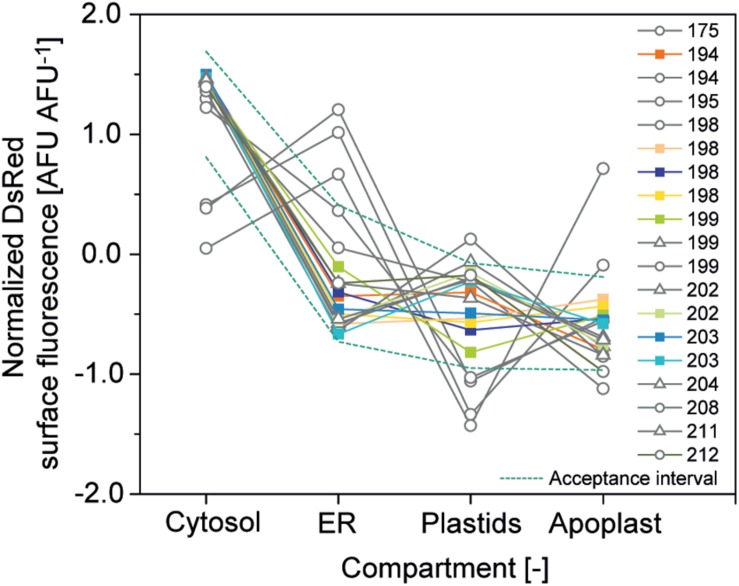
Relative DsRed accumulation in plant cell compartments. Normalized accumulation in cytosol, ER, plastids and apoplast was matched against an acceptance corridor (dotted lines) to identify high-quality cell batches. Batches meeting all quality criteria are represented by colored squares whereas gray circles and triangles indicate runs rejected due to an aberrant accumulation profile in the compartments or due to a CV > 10%, respectively.

The second criterion was a CV <10% for compartment-specific accumulation of DsRed to ensure precision and the third was relative expression of DsRed in the four compartments within a corridor of ±1 SD around the average normalized accumulation (Figure 6). The normalized accumulation in the four compartments was calculated for each batch of cells by first subtracting the grand mean from each compartment-specific mean and dividing it by the SD of the means (Equation 2). Next, we calculated the average normalized mean for each compartment along with its SD (Equation 3) and used these two values to calculate the corridor (Equation 4) (Vidakovic, 2017).

(2) $${\bar{x}}_{n\,o\,r\,m,i,j}=\frac{{\bar{x}}_{i,j}-{\bar{x}}_{j}}{\sigma_{j}}$$

(3) $${\bar{x}}_{n\,o\,r\,m,i}=\frac{\sum_{1}^{j}{\bar{x}}_{n\,o\,r\,m,i,j}}{j};\sigma_{n\,o\,r\,m,i}$$

(4) $$=\sqrt{\frac{1}{j-1}\,\sum_{1}^{j}{\left({\bar{x}}_{n\,o\,r\,m,i.j}-{\bar{x}}_{n\,o\,r\,m,i}\right)}^{2}}$$

(5) $$x_{l\,o\,w,i}={\bar{x}}_{n\,o\,r\,m,i}-\sigma_{n\,o\,r\,m,i};x_{h\,i\,g\,h,i}={\bar{x}}_{n\,o\,r\,m,i}+\sigma_{n\,o\,r\,m,i}$$

Based on these criteria ∼50% of the cell batches qualified for analysis and our efforts will focus in the future to increase cell culture quality through improved monitoring and routine inoculation from cryo-stocks to a restore defined cell state.

### Automation and Process Cost Analysis

**FIGURE 7:**
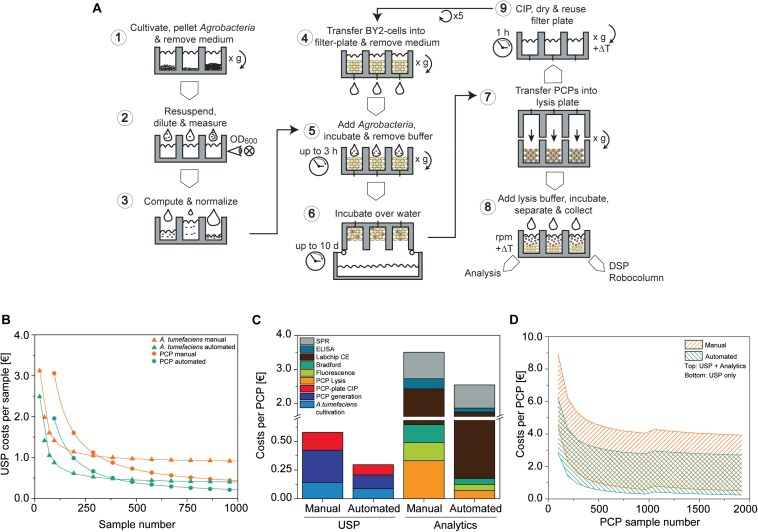
PCP automation process flow and comparison to manual preparation costs. **(A)** PCP preparation comprising *A. tumefaciens* cultivation and preparation of infiltration buffer (1–3), the generation, infiltration, incubation (4–6), and extraction of PCPs (7, 8), and final cleaning in place (CIP) (9). **(B)** Upstream production (USP) cost function in € per sample for manual and automated processing of *A. tumefaciens* (cultivation and preparation of infiltration buffer; start sample number *n*_0__*A.t.*_ = 24) and PCPs (generation and infiltration, start sample number *n*_0 PCP_ = 96). Data were fitted to a Hill function to determine costs for a large number of samples [$y=S\,T\,A\,R\,T+\left(E\,N\,D-S\,T\,A\,R\,T\right)\,\frac{x^{n}}{k^{n}+x^{n}}$, adjusted *R*^2^ = 1]. **(C)** Cost composition per PCP in € for upstream production and analytics for the manual and automated approaches (*A. tumefaciens* samples = 96, PCP samples = 960). **(D)** Cost range of the manual and automated approaches in € per PCP according to the number of PCP samples, calculated for upstream production (lower solid line) or upstream production and analytics (upper solid line). For PCP sample numbers ≤960 and >960, the *A. tumefaciens* sample numbers were 96 and 192, respectively. CE, capillary electrophoresis; DSP, downstream process; ELISA, enzyme-linked immunosorbent assay; SPR, surface plasmon resonance.

In previous sections, we showed how the cultivation of *A. tumefaciens*, PCP preparation and infiltration, and protein extraction can be automated on a liquid-handling station by only using pipetting, heating, shaking and centrifugation for PCP casting and manipulation (Figure 7A). The process flow was scripted to facilitate a read-in of typical statistical design tables, for example to test expression constructs and replicates as well as different infiltration and lysis buffer compositions, and included cross-check procedures such as barcoding to ensure technical robustness.

We compared the cost of running this automated setup with the manual PCP approach assuming that the same operations (e.g., centrifugation for PCP casting) and advanced equipment (such as manual multi-well pipettes) can be used. Costs were calculated taking into account labor, chemicals, consumables, including tips and plates, as well as energy consumption, starting with continuous BY-2 fermentation and ending with the delivery of raw data. First we calculated the upstream production (USP) costs, which rapidly decreased over the first ∼1000 samples for both *A. tumefaciens* (the PCP sample number was kept constant at 96) and PCPs (the *A. tumefaciens* sample number was kept constant at 96) (Figure 7B). *A. tumefaciens* cultivation costs eventually leveled out at 0.87 or 0.36 € for the manual and automated approaches, respectively. We defined the sample number that exhibited a deviation <5% from these values as stability threshold *x*_0_._05__, A.t._, meaning that the costs did not change considerably as the sample number increased beyond this point. For the automated and manual setup, the *n*_0_._05__, A.t._ values were 2276 and 1095, respectively. We next used *n*_0_._05__, A.t_ = 2276 as an input variable to generate the resulting cost function for PCP generation, infiltration and plate cleaning, which leveled out at 0.20 and 0.10 € and determined the *n*_0_._05__,PCP_ values 231,264 and 87,360 for the manual and automated approaches, respectively. Accordingly, the automated process required ∼60% fewer samples to achieve cost stability compared to the manual approach, at which point the automated process was also ∼54% less expensive.

Next we analyzed the cost composition for typical analytics used to evaluate PCP expression experiments [PCP lysis, extract fluorescence measurement, Bradford TSP analysis, Labchip capillary electrophoresis, enzyme-linked immunosorbent assay (ELISA), surface plasmon resonance (SPR) spectroscopy]. To better illustrate the scenario of a more frequent small-scale screening project, we reduced *n*_*A.t.*_ to 96 and n_*PCP*_ to 960 samples. The maximum USP costs for the automated approach were ∼51% relative to manual handling, and likewise the automated analytics costs were ∼68% relative to manual handling. The cost ratio of labor to consumables for manual USP was 7.5 € €^–1^, whereas cost-intensive materials such as capillary electrophoresis consumables and SPR chips decreased that ratio to 0.46 € €^–1^ in analytics, which limited the cost saving effects of automation (Figure 7C). Overall costs per PCP were strongly influenced by the number of PCP plates that were analyzed and the complexity of the analytical method. In a single-factor screening project, such as the identification of an optimal expression vector among 192 *A. tumefaciens* cultures, 1920 PCPs would be required to detect a difference in accumulation (delta) equal to the target CV of ∼5% defined above at a significance level of α = 0.05 when using ANOVA with a *post hoc* Bonferroni test for means comparison (McHugh, 2011). With this setup, the costs were 0.50 € per PCP for the manual approach but only 0.24 € per PCP for the automated setup, therefore meeting our pre-defined goal of 0.30 € per PCP. If we consider the screening of promoter libraries (Fan et al., 2012; Dey et al., 2015), the costs increase to 2.7 and 3.9 € per PCP for the automated and manual systems respectively, when a full package of analytics is included in the calculation (PCP lysis, extract fluorescence measurement, Bradford TSP analysis, Labchip capillary electrophoresis, ELISA, SPR assay). The PCP costs were also subject to fluctuations of up to 0.13 or 0.23 € per PCP for the automated and manual systems respectively, due to threshold costs for re-stocking the equipment after a given number of plates. This number was dependent on the nature of the analytical assays and the mode of operation (manual or automated) (Figure 7D). The current automated setup achieved a throughput of 4800 samples per day for an investment of ∼300,000 €. The breakeven point between the automated and manual systems will occur after 92 or 51 weeks, assuming a capacity utilization of 50 or 90%, respectively, after which the automated system becomes more cost efficient.

Finally, automation reduces the likelihood of human errors, increases throughput capacity and facilitates seamless sample tracking from gene cloning to product analysis. These advantages and potential associated cost savings are difficult to quantify and have not been accounted for in our calculation. In the future, we will increase the degree of automation, for example by directly feeding BY-2 cells from a reactor into the liquid-handling station and by feeding fresh plates from entry modules and subsequent transfer to post-infiltration incubators. We estimate, based on the current work load, that cost savings of up to ∼60% per PCP can be achieved with an additional investment of 180,000 €. Projecting this to an increased capacity of ∼9,600 samples per day, the breakeven point for additional PCP automation investments compared to the standard automation setup would shift to 312 weeks at 50% capacity utilization or 174 weeks at 90% capacity utilization (Supplementary Figure S7).

## Conclusion

Overall, our automated PCP-based transient expression protocol is designed to achieve maximum flexibility and to allow for user-friendly adjustments and selective analytics in an academic research environment. The improvements described herein achieve a CV <5% for transient expression in plant cells using high-throughput methods, reduce the costs compared to manual handling by ∼54%, while facilitating automated documentation and sample tracking to fulfill the challenges of routine protein expression screening.

## Data Availability Statement

The raw data supporting the conclusions of this article will be made available by the authors, without undue reservation, to any qualified researcher.

## Author Contributions

BG was responsible for the design and technical execution of the experiments and automation, including the protocol and custom labware design, as well as programming and manuscript writing. PO cloned the constructs used for the sub-cultivation experiments. JB planned and designed parts of the experiments, analyzed the data, and revised the manuscript.

## Conflict of Interest

The authors declare that the research was conducted in the absence of any commercial or financial relationships that could be construed as a potential conflict of interest.

## Abbreviations

AFU: arbitrary fluorescence unit
BY-2: Bright Yellow 2
CV: coefficient of variation
DOE: design of experiment
ER: endoplasmic reticulum
EDTA: ethylenediaminetetraacetic acid
OD: optical density
PCP: plant-cell pack
RWC: relative water content
SD: standard deviation
SDS: sodium dodecylsulfate
SPR: surface plasmon resonance
TSP: total soluble protein
USP: upstream production
UTR: untranslated region
WVTR: water vapor transmission rate.
