# Supplementary material for: Robot Cookies – Plant Cell Packs as an Automated High-Throughput Screening Platform Based on Transient Expression
Source: Front Bioeng Biotechnol. 2020 May 5;8:393. doi: 10.3389/fbioe.2020.00393 (PMC7214789; doi:10.3389/fbioe.2020.00393)
Supplement: Supplementary file 1 [file Data_Sheet_1.PDF]

Supplementary materials to manuscript "Robot cookies – plant cell packs as an automated high-throughput screening platform based on transient expression"

Table S1: Expression vectors used to assess the performance of process steps during automated PCP generation and infiltration**.**

Name	5′UTR	Signal peptide	Encoded protein	UniProt ID	C-terminal tag / signal	Target compartment
000001	CHS	None	IL6 (50% GC)	P05231	His	Cytosol
000002	CHS	LPH	IL6 (50% GC)	P05231	His	Apoplast
000003	CHS	TP	IL6 (50% GC)	P05231	His	Plastids
000004	CHS	LPH	IL6 (50% GC)	P05231	His, KDEL	ER
000005	omega	None	IL6 (50% GC)	P05231	His	Cytosol
000006	omega	LPH	IL6 (50% GC)	P05231	His	Apoplast
000007	omega	TP	IL6 (50% GC)	P05231	His	Plastids
000008	omega	LPH	IL6 (50% GC)	P05231	His, KDEL	ER
000009	TL	None	IL6 (50% GC)	P05231	His	Cytosol
000010	TL	LPH	IL6 (50% GC)	P05231	His	Apoplast
000011	TL	TP	IL6 (50% GC)	P05231	His	Plastids
000012	TL	LPH	IL6 (50% GC)	P05231	His, KDEL	ER
000013	CHS	None	IL6 (33% GC)	P05231	His	Cytosol
000014	CHS	LPH	IL6 (33% GC)	P05231	His	Apoplast
000015	CHS	TP	IL6 (33% GC)	P05231	His	Plastids
000016	CHS	LPH	IL6 (33% GC)	P05231	His, KDEL	ER
000017	omega	None	IL6 (33% GC)	P05231	His	Cytosol

Table S1 continued.

Name	5′UTR	Signal peptide	Encoded protein	UniProt ID	C-terminal tag / signal	Target compartment
000018	omega	LPH	IL6 (33% GC)	P05231	His	Apoplast
000019	omega	TP	IL6 (33% GC)	P05231	His	Plastids
000020	omega	LPH	IL6 (33% GC)	P05231	His, KDEL	ER
000021	TL	None	IL6 (33% GC)	P05231	His	Cytosol
000022	TL	LPH	IL6 (33% GC)	P05231	His	Apoplast
000023	TL	TP	IL6 (33% GC)	P05231	His	Plastids
000024	TL	LPH	IL6 (33% GC)	P05231	His, KDEL	ER
000025	CHS	None	IL8 (50% GC)	P10145	His	Cytosol
000026	CHS	LPH	IL8 (50% GC)	P10145	His	Apoplast
000027	CHS	TP	IL8 (50% GC)	P10145	His	Plastids
000028	CHS	LPH	IL8 (50% GC)	P10145	His, KDEL	ER
000029	omega	None	IL8 (50% GC)	P10145	His	Cytosol
000030	omega	LPH	IL8 (50% GC)	P10145	His	Apoplast
000031	omega	TP	IL8 (50% GC)	P10145	His	Plastids
000032	omega	LPH	IL8 (50% GC)	P10145	His, KDEL	ER
000033	TL	None	IL8 (50% GC)	P10145	His	Cytosol
000034	TL	LPH	IL8 (50% GC)	P10145	His	Apoplast

Table S1 continued.

Name	5′UTR	Signal peptide	Encoded protein	UniProt ID	C-terminal tag / signal	Target compartment
000035	TL	TP	IL8 (50% GC)	P10145	His	Plastids
000036	TL	LPH	IL8 (50% GC)	P10145	His, KDEL	ER
000037	CHS	None	IL8 (33% GC)	P10145	His	Cytosol
000038	CHS	LPH	IL8 (33% GC)	P10145	His	Apoplast
000039	CHS	TP	IL8 (33% GC)	P10145	His	Plastids
000040	CHS	LPH	IL8 (33% GC)	P10145	His, KDEL	ER
000041	omega	None	IL8 (33% GC)	P10145	His	Cytosol
000042	omega	LPH	IL8 (33% GC)	P10145	His	Apoplast
000043	omega	TP	IL8 (33% GC)	P10145	His	Plastids
000044	omega	LPH	IL8 (33% GC)	P10145	His, KDEL	ER
000045	TL	None	IL8 (33% GC)	P10145	His	Cytosol
000046	TL	LPH	IL8 (33% GC)	P10145	His	Apoplast
000047	TL	TP	IL8 (33% GC)	P10145	His	Plastids
000048	TL	LPH	IL8 (33% GC)	P10145	His, KDEL	ER
000049	CHS	None	PPKRO	None	His	Cytosol
000050	CHS	LPH	PPKRO	None	His	Apoplast
000051	CHS	TP	PPKRO	None	His	Plastids
000052	CHS	LPH	PPKRO	None	His, KDEL	ER
000053	omega	None	PPKRO	None	His	Cytosol
000054	omega	LPH	PPKRO	None	His	Apoplast

Table S1 continued.

Name	5′UTR	Signal peptide	Encoded protein	UniProt ID	C-terminal tag / signal	Target compartment
000055	omega	TP	PPKRO	None	His	Plastids
000056	omega	LPH	PPKRO	None	His, KDEL	ER
000057	TL	None	PPKRO	None	His	Cytosol
000058	TL	LPH	PPKRO	None	His	Apoplast
000059	TL	TP	PPKRO	None	His	Plastids
000060	TL	LPH	PPKRO	None	His, KDEL	ER
000061	CHS	His	PPKRO	None	None	Cytosol
000062	CHS	LPH, His	PPKRO	None	None	Apoplast
000063	CHS	TP, His	PPKRO	None	None	Plastids
000064	CHS	LPH, His	PPKRO	None	KDEL	ER
000065	omega	His	PPKRO	None	None	Cytosol
000066	omega	LPH, His	PPKRO	None	None	Apoplast
000067	omega	TP, His	PPKRO	None	None	Plastids
000068	omega	LPH, His	PPKRO	None	KDEL	ER
000069	TL	His	PPKRO	None	None	Cytosol
000070	TL	LPH, His	PPKRO	None	None	Apoplast
000071	TL	TP, His	PPKRO	None	None	Plastids
000072	TL	LPH, His	PPKRO	None	KDEL	ER
000101	CHS	None	DsRed	Q9U6Y8	His	Cytosol
000102	CHS	LPH	DsRed	Q9U6Y8	His	Apoplast
000103	CHS	LPH	DsRed	Q9U6Y8	His, KDEL	ER
000104	CHS	TP	DsRed	Q9U6Y8	His	Plastids
000137	CHS[1,2]	LPH[1], TP[2]	2F5 HC/LC[1], DsRed[2]	anti-A0A3G1DXK7[1], Q9U6Y8[2]	None	Apoplast[1], Plastids[2]
000189	CHS	None	P19	P11690	None	Cytosol

2F5 HC/LC – anti-envelope glycoprotein gp160 from Human immunodeficiency virus 1 monoclonal antibody heavy and light chain; CHS – *Petroselinum hortense* chalcone synthase; DsRed – fluorescent protein from *Discosoma* sp. (sea anemone); ER – endoplasmic reticulum; GC – guanine-cytosine content of the DNA coding sequence; His – hexahistidine; IL6 – IL8 – human interleukin 6/8; KDEL – sequence for protein retention in the endoplasmic reticulum; LPH – signal peptide from the heavy chain of the murine Tobacco mosaic virus-specific monoclonal antibody 24; omega – omega sequence of Tobacco mosaic virus; P19 – 19 kDa protein from Tomato bushy stunt virus; PPKRO – polyphosphate kinase from *Rhodococcus opacus*; TP – transit peptide from the ribulose-1,5-bisphosphate carboxylase/oxygenase small subunit of *Solanum tuberosum*; [1 –2] – expression cassette 1 and 2.

Table S2: Design summary and analysis of variance for PCP generation by centrifugation DOE approach.

**Design Summary**							
FileVersion	10.0.0.3						
StudyType	ResponseSurface	Subtype	Randomized				
DesignType	HistoricalData	Runs	88				
DesignModel	Quadratic	Blocks	No Blocks				
Factor	Name	Units	Type	Subtype	Min	Max	Levels
A	Time	[minutes]	Numeric	Continuous	1	10	according to base model
B	Centrifugal force	[× g]	Numeric	Continuous	451	3162	according to base model

**Response 1**	**PCP density [g cm³]**
Transform:	None

Source	df	F-Value	p-value
Model	7	114.386	<0.001
A-Time	1	13.953	<0.001
B-Centrifugal force	1	12.485	0.001
AB	1	1.081	<0.001
A^2	1	11.122	0.001
B^2	1	23.489	<0.001
AB^2	1	5.553	0.021
B^3	1	31.285	<0.001
Lack of Fit	3	1.274	0.289
R^2	0.909		
Adjusted R^2	0.901		
Predicted R^2	0.892		

df – degrees of freedom.

Table S3: Design summary and analysis of variance of PCP tilt and infiltration suspension removal DOE approach.

**Design Summary**							
File Version	10.0.0.3						
Study Type	Response Surface	Subtype	Randomized				
Design Type	Historical Data	Runs	576				
Design Model	Quadratic	Blocks	No Blocks				
Factor	Name	Units	Type	Subtype	Min	Max	Levels
A	PCP tilt	[°]	Numeric	Discrete	2.9	35.1	6
B	Centrifugal force	[× g]	Numeric	Discrete	100	1800	7
C	Compartment	[-]	Categoric	Nominal	Cytosol	Apoplast	2

**Response 1**	**PCP DsRed surface fluorescence [AFU]**
Transformation	None

Source	df	F-Value	p-value
Model	18	2429.444	<0.001
A-PCP tilt	1	11.967	0.001
B-Centrifugal force	1	2105.505	<0.001
C-Compartment	1	12095.017	<0.001
AB	1	15.489	<0.001
AC	1	0.084	0.772
BC	1	628.892	<0.001
A^2	1	2.964	0.086
B^2	1	136.988	<0.001
ABC	1	10.389	0.001
A^2B	1	0.057	0.811
AB^2	1	2.473	0.116
B^2C	1	1750.367	<0.001
A^3	1	12.170	0.001
B^3	1	6520.289	<0.001
A^2B^2	1	5.753	0.017
B^3C	1	1359.575	<0.001
A^4	1	8.589	0.004
B^4	1	892.940	<0.001
Lack of Fit	41	19.902	<0.001
R^2	0.990		
Adjusted R^2	0.989		
Predicted R^2	0.989		

Table S3 continued.

**Response 2**	**DsRed expression [mg kg^{-1}]**		
Transformation	None		
Source	df	F-Value	p-value
Model	17	1113.377	<0.001
A-PCPtilt	1	6.188	0.013
B-Centrifugal force	1	161.129	<0.001
C-Compartment	1	4718.454	<0.001
AB	1	12.734	<0.001
AC	1	26.542	<0.001
BC	1	65.713	<0.001
A^2	1	2.854	0.092
B^2	1	2.141	0.144
ABC	1	8.255	0.004
A^2C	1	6.102	0.014
AB^2	1	0.340	0.560
B^2C	1	1025.110	<0.001
A^3	1	5.713	0.017
B^3	1	1147.920	<0.001
AB^3	1	14.198	<0.001
B^3C	1	339.710	<0.001
B^4	1	77.512	<0.001
Lack of Fit	42	3.871	<0.001
R^2	0.977		
Adjusted R^2	0.976		
Predicted R^2	0.975		

Table S3 continued.

**Response 3**	**Average CV of DsRed expression [%]**		
Transformation	None		
Source	df	F-Value	p-value
Model	8	230678.867	<0.001
B-Centrifugal force	1	5810.713	<0.001
C-Compartment	1	357.792	<0.001
BC	1	316.188	<0.001
B^2	1	296.327	<0.001
BC	1	3328.011	<0.001
B^3	1	183100.950	<0.001
B^3C	1	2006.382	<0.001
B^4	1	24836.093	<0.001
Lack of Fit	51		
R^2	1.000		
Adjusted R^2	1.000		
Predicted R^2	1.000		

df – degrees of freedom.

Table S4: Design summary and analysis of variance of membrane-based PCP incubation DOE approach.

**Design Summary**							
File Version	10.0.0.3						
Study Type	Response Surface	Subtype	Randomized				
Design Type	Historical Data	Runs	24				
Design Model	Quadratic	Blocks	No Blocks				
Factor	Name	Unit	Type	Subtype	Min	Max	Levels
A	Water reservoir volume	[µL]	Numeric	Discrete	0	100	2
B	Covered surface area (lid)	[%]	Numeric	Discrete	0	100	2
C	Logit Membrane WVTR	[g m^2 d^{-1}]	Numeric	Discrete	-8.343	8.343	5

**Response 1**	**Average mass loss in plate [% of start mass]**		
Transformation	None		
Source	df	F-Value	p-value
Model	3	25.994	<0.001
B-Covered surface area (lid)	1	31.962	<0.001
C-Logit Membrane WVTR	1	30.319	<0.001
BC	1	18.941	<0.001
Lack of Fit	16	1.020	0.554
R^2	0.796		
Adjusted R^2	0.765		
Predicted R^2	0.735		

**Response 2**	**Average DsRed surface fluorescence [AFU]**		
Data transformation:	None		
Source	df	F-Value	p-value
Model	4	17.721	<0.001
B-Covered surface area (lid)	1	7.422	0.013
C-Logit Membrane WVTR	1	10.468	0.004
BC	1	43.484	<0.001
C^2	1	9.547	0.006
Lack of Fit	15	6.398	0.043
R^2	0.789		
Adjusted R^2	0.744		
Predicted R^2	0.687		

Table S4 continued.

**Response 3**	**Mass loss ratio inner outer wells [-]**		
Transformation	None		
Source	df	F-Value	p-value
Model	5	10.126	<0.001
A-Water reservoir volume	1	1.264	0.276
B-Covered surface area (lid)	1	0.174	0.681
C-Logit Membrane WVTR	1	38.334	<0.001
AB	1	5.502	0.031
BC	1	3.561	0.075
Lack of Fit	14	1.894	0.283
R^2	0.738		
Adjusted R^2	0.665		
Predicted R^2	0.490		

Factor C - Water vapor transmission rate values of membranes (1; 450; 700; 4200; 4201) covering the PCP incubation plate were logit transformed prior to analysis – using $y'=\ln((y-0)/(4202-y))$; AFU – arbitrary fluorescence units; df – degrees of freedom; WVTR – water vapor transmission rate.

Table S5: Design summary and analysis of variance of chemical PCP lysis DOE approach.

**Design Summary**							
File Version	10.0.0.3						
Study Type	Response Surface	Subtype	Randomized				
Design Type	I-optimal	Coordinate Exchange	Runs	41			
Design Model	Reduced Cubic	Blocks	No Blocks				
Factor	Name	Units	Type	Subtype	Min	Max	Levels
A	SDS	[% (m v^{-1})]	Numeric	Continuous	-3	0	according to base model
B	pH	[-]	Numeric	Discrete	7	9	3
C	Temperature	[°C]	Numeric	Discrete	37	60	3
D	EDTA	[mM]	Numeric	Continuous	0	20	according to base model
E	Compartment	[-]	Categoric	Nominal	Cytosol	Plastid	4

**Response 1**	**DsRed concentration [mg kg^{-1}]**		
Transformation	None		
Source	df	F-Value	p-value
Model	18	20.837	<0.001
A-SDS	1	0.132	0.720
B-pH	1	0.236	0.632
C-Temperature	1	40.784	<0.001
D-EDTA	1	38.669	<0.001
E-Compartment	3	28.968	<0.001
AB	1	19.146	<0.001
CD	1	37.827	<0.001
CE	3	9.888	<0.001
DE	3	14.407	<0.001
A^2	1	8.974	0.007
C^2	1	14.976	0.001
D^2	1	10.468	0.004
Lack of Fit	17	0.584	0.814
R^2	0.945		
Adjusted R^2	0.899		
Predicted R^2	0.812		

Table S5 continued.

**Response 2**	**Total soluble protein [mg kg^{-1}]**		
Transformation	Base 10 Log	Constant:	0
Source	df	F-Value	p-value
Model	5	44.874	<0.001
A-SDS	1	200.578	<0.001
C-Temperature	1	0.857	0.361
D-EDTA	1	10.130	0.003
AC	1	12.031	0.001
CD	1	16.031	<0.001
Lack of Fit	30	7.572	0.016
R^2	0.865		
Adjusted R^2	0.846		
Predicted R^2	0.826		

**Response 3**	**DsRed TSP^{-1} [kg kg^{-1}]**		
Transform:	Base 10 Log	Constant:	0
Source	df	F-Value	p-value
Model	10	7.362	<0.001
A-SDS	1	32.023	<0.001
B-pH	1	1.437	0.240
C-Temperature	1	1.957	0.172
D-EDTA	1	9.106	0.005
E-Compartment	3	4.324	0.012
CE	3	2.937	0.049
Lack of Fit	25	96.028	<0.001
R^2	0.710		
Adjusted R^2	0.614		
Predicted R^2	0.451		

Factor A – SDS concentration range (0.001–1.0% (m v^{-1})) was log transformed prior to model building; df – degrees of freedom; TSP – total soluble protein.

Table S6: Comparison of DoE model predictions and confirmation experiments.

DoE	Design and model description	Response	Model prediction at relevant location in the design[a]	Value obtained in confirmation experiment[d]
Centrifugation	Table S2	PCP density [g cm^3]	0.54 ± 0.02 (n=77)	0.56 ± 0.01 (n=6)
Tilt surface[a]	Table S3	PCP DsRed surface fluorescence [AFU]	34339 ± 1653 (n = 415)	38292 ± 916 (n = 16)
		DsRed expression [mg kg^{-1}]	130.8 ± 9.2 (n = 413)	272.3 ± 17.0 (n = 16)
		Average CV of DsRed expression [%]	5.4 ± 0.5 (n = 415)	6.3 ± 0.4 (n = 16)
Membrane coverage[a]	Table S4	Average mass loss in plate [% of start mass]	79 ±14 (n = 4)	0.53[c]
		Average relative DsRed surface fluorescence [AFU AFU^{-1}]	1.04 ±0.31 (n = 4)	0.98±0.12 (n = 40)
		Mass loss ratio inner outer wells [-]	0.86 ±0.08 (n = 4)	0.72[c]
PCP lysis[a]	Table S5	DsRed concentration [mg kg^{-1}]	45.4 ± 14.2 (n = 4)	59.2 ± 2.3 (n = 6)
		Total soluble protein [mg kg^{-1}]	656.5 ± 180.0 (n = 5)	1257.2 ± 388.3 (n = 6)
		DsRed TSP^{-1} [kg kg^{-1}]	0.043 ± 0.033 (n = 5)	0.051 ± 0.017 (n = 6)

[a] – values for cytosolic expression; [b] – values for membrane with WVTR of 4200 [g m^{-2} d^{-1}]; [c] – ratio of n=8 (inner) and n=16 (peripheral) values, see Fig. S2; [d] – relative to highest signal in experiment; values without annotation are given as $\bar{x}$ ± standard deviation, the number of replicates is reported in brackets.

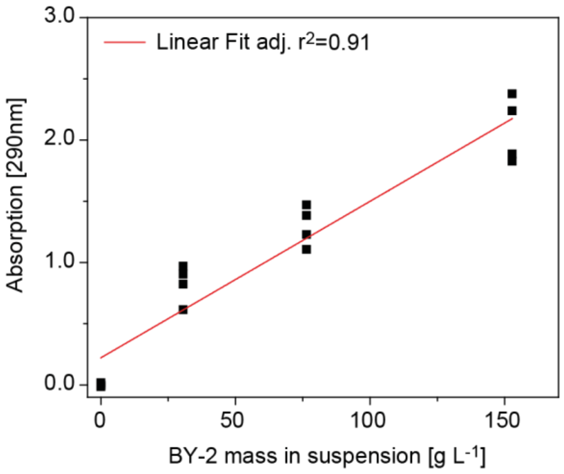

Figure S1: Standard curve of BY-2 cell mass in suspension. Pelleted BY-2 cells were resuspended in standard lysis buffer (1.0% (m v^{-1}) SDS, 50 mM EDTA, 50 mM Tris, pH 8.0) and absorption was immediately measured at 290 nm (n = 4). Red line indicates linear fit with adjusted r^2 of 0.91.

	1	2	3	4	5	6	7	8	9	10	11	12
A	P	P	P	P	P			P	P	P	P	P
B	P											P
C	P		I	I					I	I		P
D	P		I	I					I	I		P
E	P		I	I					I	I		P
F	P		I	I					I	I		P
G	P											P
H	P	P	P	P	P			P	P	P	P	P

Figure S2: Classification of peripheral and inner PCPs in a 96-well plate as used for mass ratio experiments. Inner PCPs (I), n = 8 and peripheral PCPs (P), n = 16.

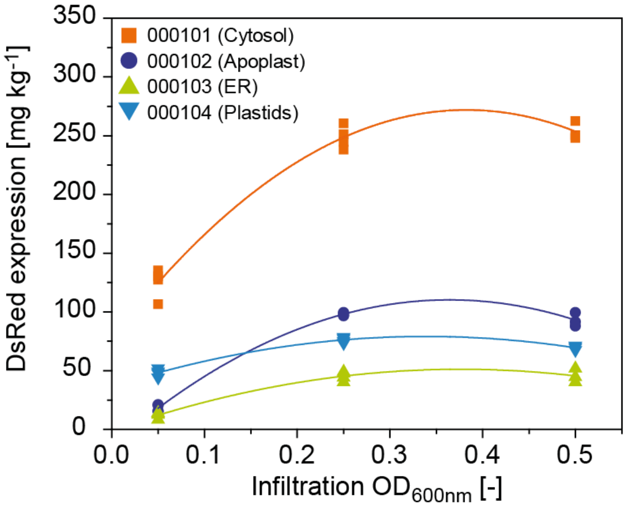

Figure S3: DsRed expression in PCPs as a function of infiltration suspension OD600nm. PCPs were infiltrated with *A. tumefaciens* infiltration suspension (OD_{600nm} of 0.05, 0.25, or 0.5) harboring vectors 000101, 000102, 000103, or 000104 for expression of DsRed in the cytosol, apoplast, ER or plastids, respectively. PCPs were incubated for 3 days prior to extraction with n = 4 per treatment. Second-order polynomial curves were fitted to data points with adjusted r^2 values of 0.974, 0.992, 0.943, and 0.961 for cytosol, apoplast, ER and plastids, respectively.

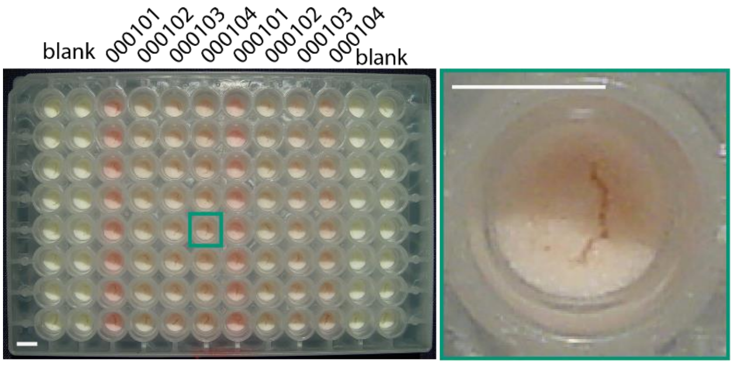

Figure S4: Crack formation in PCPs generated by vacuum. DsRed was expressed from constructs 000101 (columns 3 and 7), 000102 (columns 4 and 8), 000103 (columns 5 and 9), or 000104 (columns 6 and 10) in the cytosol, apoplast, ER or plastids, respectively. Blanks were prepared in columns 1 and 2, and 11 and 12. Picture was taken after incubation for 3 days. Green box shows enlargement of well E6. White scale bar = 5 mm.

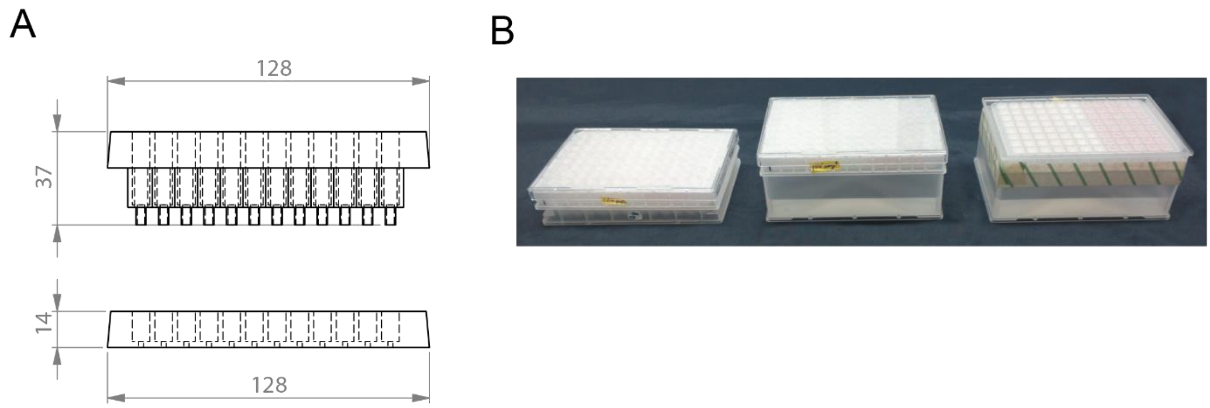

Figure S5: High and low profile 96-well filter plates for PCP generation and incubation. (A) Receiver Plate with a pore size of 50 µm (top) and an AgroPrep Advance PP/PE 30–40 µm filter plate. Dimensions in mm. **(B)** PCP incubation with membrane and lid over a water reservoir with 150 µL per well (left), with membrane and lid over 1-well water reservoir with 150 mL per plate (mid), or inverted over 1-well water reservoir with 150 mL per plate (right).

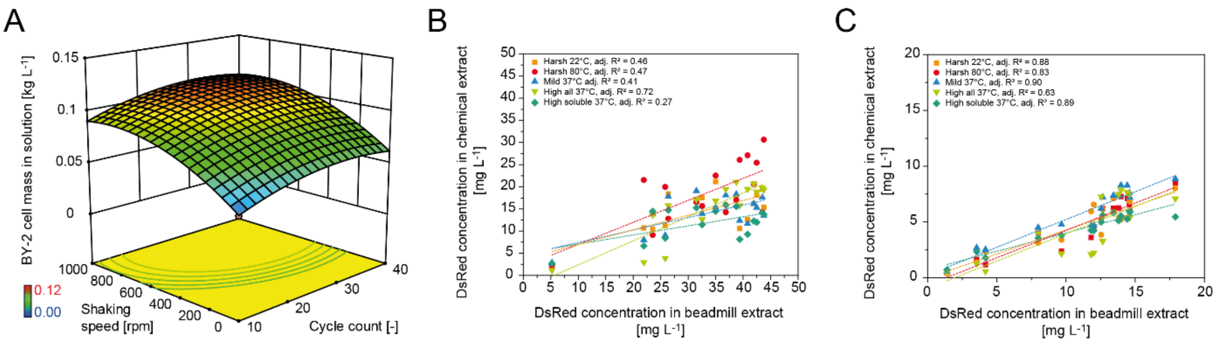

Figure S6: Design of experiments for disruption of PCPs in chemical lysis buffer and extraction correlation curves for cytosolic and ER localized DsRed. **(A)** Response surface of BY-2 cell mass in suspension [kg L^{-1}] as a function of orbital shaking speed (rpm) with 4 mm eccentricity and pipetting cycle count. DsRed concentration in the lysis buffer [mg L^{-1}] showing comparison between chemical lysis and mechanical reference extraction by bead mill, starting with PCPs expressing DsRed in the cytosol **(B)** (vector 000101) and in the ER **(C)** (vector 000103) with linear fits as dotted lines.

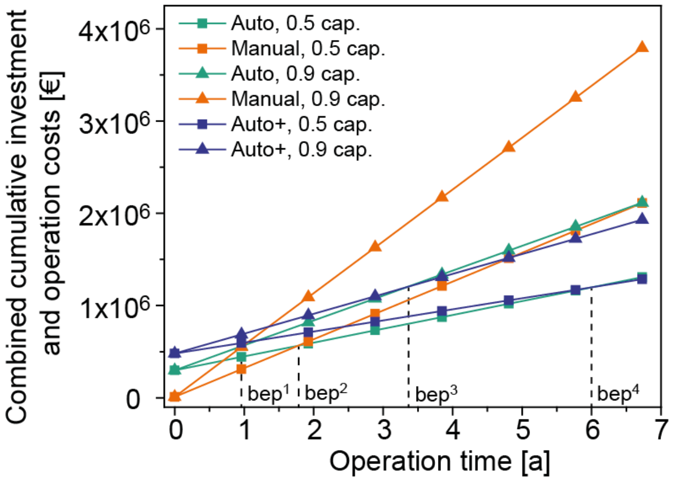

Figure S7: Breakeven points of costs for the manual and automated PCP protocol. The automated protocol requires an investment of 300,000 € for a basic system (Auto, 24,000 samples per week) or 480,000 € considering a higher degree of automation (Auto+, 48,000 samples per week), with costs of 0.24 and 0.096 € per PCP, respectively. Investments for the manual protocol (Man, 24,000 samples week^{-1}) are 11,800 € with costs of 0.50 € per PCP. Costs were calculated for a capacity utilization of either 50% or 90%. Intersections of lines indicate breakeven points of manual and standard automation at 50% (bep^1) and 90% (bep^2) capacity utilization, as well as of standard an augmented automation at 50% (bep^3) and 90% (bep^4).
